# Bowman’s Practical Chemistry

**Published:** 1849-05

**Authors:** 


### BOWMAN'S PRACTICAL CHEMISTRY.

The object of this work is to explain and render simple to the beginner the various processes employed in analysis, or which illustrate the principles of chemical science. In this respect it has claims upon students in search of a practical knowledge of chemistry, superior to any work on the subject extant in our language. Its object is to instruct the novice in the operations of the laboratory, as well as in their rationale, and its aim has been so well fulfilled that we hardly see how the student could wish a more satisfactory guide. Not only to students of medicine, who wish to acquire some skill in chemical manipulation, but to the apprentices of apothecaries and druggists it is a manual which will prove exceedingly serviceable. A copy of it ought to be found in every drug-store in the country.

